# Predictors of anemia in women of reproductive age: Biomarkers Reflecting Inflammation and Nutritional Determinants of Anemia (BRINDA) project

**DOI:** 10.3945/ajcn.116.143073

**Published:** 2017-06-14

**Authors:** James P Wirth, Bradley A Woodruff, Reina Engle-Stone, Sorrel ML Namaste, Victor J Temple, Nicolai Petry, Barbara Macdonald, Parminder S Suchdev, Fabian Rohner, Grant J Aaron

**Affiliations:** 1 GroundWork, Fläsch, Switzerland;; 2 Department of Nutrition, University of California, Davis, CA;; 3 Strengthening Partnerships, Results, and Innovations in Nutrition Globally, Arlington, VA;; 4 Helen Keller International, New York City, NY;; 5 *Eunice Kennedy Shriver* National Institute of Child Health and Human Development, NIH, Bethesda, MD;; 6 School of Medicine and Health Sciences, University of Papua New Guinea, Port Moresby, Papua New Guinea;; 7 Canadian Foodgrains Bank, Winnipeg, Canada;; 8 Nutrition Branch, Centers for Disease Control and Prevention, Atlanta, GA;; 9 Department of Pediatrics, Emory University, Atlanta, GA; and; 10 Global Alliance for Improved Nutrition, Geneva, Switzerland

**Keywords:** anemia, determinants, inflammation, iron, malaria, micronutrient deficiencies, risk factors, women, women of reproductive age

## Abstract

**Background:** Anemia in women of reproductive age (WRA) (age range: 15–49 y) remains a public health problem globally, and reducing anemia in women by 50% by 2025 is a goal of the World Health Assembly.

**Objective:** We assessed the associations between anemia and multiple proximal risk factors (e.g., iron and vitamin A deficiencies, inflammation, malaria, and body mass index) and distal risk factors (e.g., education status, household sanitation and hygiene, and urban or rural residence) in nonpregnant WRA.

**Design:** Cross-sectional, nationally representative data from 10 surveys (*n* = 27,018) from the Biomarkers Reflecting Inflammation and Nutritional Determinants of Anemia (BRINDA) project were analyzed individually and pooled by the infection burden and risk in the country. We examined the severity of anemia and measured the bivariate associations between anemia and factors at the country level and by infection burden, which we classified with the use of the national prevalences of malaria, HIV, schistosomiasis, sanitation, and water-quality indicators. Pooled multivariate logistic regression models were constructed for each infection-burden category to identify independent determinants of anemia (hemoglobin concertation <120 g/L).

**Results:** Anemia prevalence was ∼40% in countries with a high infection burden and 12% and 7% in countries with moderate and low infection burdens, respectively. Iron deficiency was consistently associated with anemia in multivariate models, but the proportion of anemic women who were iron deficient was considerably lower in the high-infection group (35%) than in the moderate- and low-infection groups (65% and 71%, respectively). In the multivariate analysis, inflammation, vitamin A insufficiency, socioeconomic status, and age were also significantly associated with anemia, but malaria and vitamin B-12 and folate deficiencies were not.

**Conclusions:** The contribution of iron deficiency to anemia varies according to a country’s infection burden. Anemia-reduction programs for WRA can be improved by considering the underlying infection burden of the population and by assessing the overlap of micronutrient deficiencies and anemia.

## INTRODUCTION

Anemia in women of reproductive age (WRA) (age range: 15–49 y) remains a public health problem globally, and reducing anemia in WRA by 50% by 2025 is 1 of 6 global nutrition targets that have been set forth by the World Health Assembly (1). As of 2011, ∼496 million nonpregnant women were anemic (2), which is an increase of ∼50 million nonpregnant women from 1995 (3). According to a recent systematic review and meta-analysis, anemia during pregnancy increased risks of poor birth outcomes such as preterm birth, low birth weight, and perinatal and neonatal mortality (4). Maternal mortality has also been associated with hemoglobin concentrations during pregnancy (5) with risk of maternal mortality decreasing by 25% for every 10-g/L increase in hemoglobin. Moreover, anemia results in reduced work productivity in nonpregnant WRA (6–9), which is likely due to reduced oxygen-carrying capacity in an individual’s blood (10).

The Biomarkers Reflecting Inflammation and Nutritional Determinants of Anemia (BRINDA) project developed its own anemia causative framework that is presented in the methodologic overview in this supplement, which is an open access publication (11). Compared with other conceptual models (12, 13), the BRINDA anemia framework details the associations between micronutrient deficiencies, inflammation, hemoglobinopathies, and anemia. The framework also illustrates that malaria can cause anemia through the destruction of erythrocytes but does not cause iron loss (14), and exposure to unsafe water and inadequate sanitation, a lack or nonuse of bed nets, food insecurity, and household poverty (12) likely contribute to anemia by increasing the risk of infectious diseases and decreasing the availability and consumption of micronutrient-rich foods.

Iron deficiency, in particular, has long been considered a major cause of anemia in most populations. It has long been presumed that iron deficiency contributes to 50% of all anemia (15). This approximation has been frequently referenced (15–17) beginning with a simple calculation derived in 1985 under the presumption that men do not become iron deficient because they do not experience rapid growth or menstruation, but they do encounter the other risk factors for anemia (18). This approximation has been supported by findings from Stoltzfus et al. (5) and, more recently, by Stevens et al. (3) who showed that 50% of anemia in women was amenable to iron supplementation by applying hemoglobin shifts from meta-analyses of supplementation trials to new global estimates of anemia prevalence. In addition, a recent analysis that used data from a meta-analysis on the response of hemoglobin to iron-fortification studies concluded that iron deficiency was the primary contributor to anemia in multiple geographic settings (13).

Although these studies reached similar conclusions concerning the etiology of anemia, they each modeled the determinants of anemia and the proportion of anemia that were attributable to common risk factors such as iron deficiency. Multiple individual studies that have examined the determinants of anemia with the use of individual-level data on micronutrient and disease status have shown that the proportion of anemia that is attributable to iron deficiency is highly variable and that other risk factors may play more important roles in determining hemoglobin concentration and anemia (19, 20).

The objectives of this article were to assess the association between anemia and posited risk factors in nonpregnant WRA in 10 surveys. Although hemoglobin is routinely assessed in health and nutrition surveys, it is less common to have survey data that include measurements of several potential risk factors for anemia. The BRINDA project is uniquely positioned to address this knowledge gap through a secondary analysis of cross-sectional survey data that include anemia and other factors such as inflammation and micronutrient biomarkers and household-level risk factors for anemia (21).

## METHODS

We conducted these analyses with the use of data from the BRINDA project (www.BRINDA-nutrition.org); details of the project objectives and methodology have previously been published (21). The BRINDA protocol was reviewed by the institutional review boards of the NIH and was deemed to be non–human subjects research. The surveys included in the BRINDA project were nationally or regionally representative, and the inclusion criteria were as follows: *1*) surveys were conducted between 2004 and 2012; *2*) surveys included preschool children or nonpregnant WRA; and *3*) surveys measured ≥1 indicator of anemia (hemoglobin), iron (ferritin or soluble transferrin receptor), or vitamin A status [(retinol-binding protein (RBP) or retinol)] and ≥1 biomarker of inflammation [(α-1-acid glycoprotein (AGP) or C-reactive protein (CRP)] (21). In this analysis, we included surveys with measures of hemoglobin in nonpregnant WRA (age range: 15–49 y) and examined data from 10 nationally representative cross-sectional surveys from 3 countries in the Americas (Colombia, Mexico, and the United States), 3 countries in West Africa (Cameroon, Côte d’Ivoire, and Liberia), one country in Central Asia (Georgia), and 2 countries in Southeast Asia and the Pacific (Laos and Papua New Guinea). We excluded Papua New Guinea from analyses that were related to iron status because only soluble transferrin receptor and not ferritin was measured. Descriptions of the original surveys and relevant references are included in the methodologic summary in this issue (11).

### Laboratory analyses

Hemoglobin concentrations were measured with the use of a Beckman Coulter MAXM hematology flow cytometer (Beckman Coulter Inc.) in the United States or a portable hemoglobinometer in Georgia (HumaMeter; Human GmbH) and in all other countries (HemoCue; HemoCue AB). Venous or capillary blood was collected from each respondent, and plasma or serum was stored at −20°C until analysis; the Papua New Guinea survey used dried blood spots. Ferritin, RBP, CRP, and AGP concentrations were assessed with the use of a sandwich ELISA at the VitMin Laboratory in Willstaett, Germany, in 5 of 10 WRA data sets (22). CRP was measured with the use of in-country methods in Mexico (Behring Nephelometer 100 Analyzer; Behringwerke AG) and the United States (immunoassay). Serum retinol was measured via HPLC (23) in the United States. Malaria was assessed with the use of microscopy in Côte d’Ivoire (24), a Paracheck Pf rapid diagnostic test (Orchid Biomedical System) in Liberia, and the plasma histidine-rich protein 2 (Cellabs Pty Ltd.) in Cameroon. Additional information on laboratory methods is further described in the methodologic overview in this supplement (11).

### Grouping countries by infection burden

We categorized surveys into 3 groups representing risk and burden of infectious disease and inflammation (hereinafter referred to as infection burden), and carried out the data analysis separately for each group. The logic for this approach was that the causes of anemia will vary depending on environmental and socioeconomic characteristics and the intensity of exposure to infections and to inflammation-inducing conditions (e.g., obesity). Countries were assigned to infection groups by adapting the approach developed by Petry et al. (25), whereby national-level prevalence estimates of malaria, HIV infection, access to improved drinking water and sanitation facilities, and schistosomiasis were used to calculate an equally weighted infection score for each country and to group countries on the basis of their infection scores (see **Supplemental Table 1** for details). Our calculations varied from those used by Petry et al. (25) in the following 2 ways: *1*) our infection score did not include the prevalence of obesity because we aimed to group countries on the basis of characteristics that are related to infectious disease only, and *2*) the ranges used for each infection group were modified slightly because of the relatively small number of data sets that were available to the BRINDA project. With the use of this approach, Cameroon, Côte d'Ivoire, Liberia, Laos, and Papua New Guinea were classified as countries with a high infection burden; Mexico and Colombia were classified as countries with a moderate infection burden; and Georgia and the United States were classified as having a low infection burden.

### Determinants of anemia and case definitions

The WHO definitions of anemia status were used to classify anemia in nonpregnant women (26). Any anemia was defined as a hemoglobin concentration <120 g/L. Severe, moderate, and mild anemia were defined as hemoglobin concentrations <80, 80–109, and 110–119 g/L, respectively. Hemoglobin concentrations were adjusted for altitude and the intensity of cigarette smoking according to WHO procedures (26) in the Colombia, Georgia, Papua New Guinea, and Mexico 2006, and Mexico 2012 surveys. In Laos, hemoglobin was adjusted only for altitude, and in the United States, hemoglobin was adjusted only for smoking. No adjustments to hemoglobin were made in the Côte d’Ivoire, Cameroon, and Liberia surveys. Hemoglobin concentrations were considered biologically implausible when values fell outside of 40–180 g/L and were set to missing. With the use of the WHO classification of the public health significance of anemia (15), an anemia prevalence of 5.0–19.9% was used to denote a mild public health problem, a prevalence of 20.0–39.9% was used to denote a moderate problem, and a prevalence of ≥40% was used to denote a severe problem. A prevalence of <5% was considered normal.

Ferritin was used as the indicator of iron status in population-based surveys according to WHO recommendations (27). Ferritin concentrations were adjusted for inflammation as measured via AGP and CRP concentrations with the use of the internal regression correction (IRC) approaches that are defined by Namaste et al. (28) in this supplement. Briefly, ferritin was adjusted with the use of both CRP and AGP when available and with only CRP in countries that did not measure AGP. The IRC approach enabled a continuous, rather than categorical, adjustment of ferritin concentrations and resulted in a greater difference from the unadjusted prevalence derived from previous methods that adjusted ferritin when inflammation levels reached certain thresholds (29, 30). An illustrative example of adjusting ferritin for CRP and AGP with the use of the IRC approach is presented in **Supplemental Figure 1**. RBP and retinol (in the United States only) were used as measures of vitamin A status. Although RBP and retinol concentrations are affected by inflammation, the findings by Larson et al. (31) in this supplement show that the relations between these indicators and inflammatory markers in women in the BRINDA data set were weak and inconsistent. As such, we did not adjust RBP or retinol concentrations for inflammation for this analysis.

Iron deficiency was defined as an adjusted ferritin concentration <15 μg/L, which indicated depleted iron stores (27). Iron-deficiency anemia was defined as concurrent iron deficiency (i.e., a ferritin concentration <15 μg/L) and anemia (i.e., hemoglobin concentration <120 g/L). Vitamin A deficiency and vitamin A insufficiency were defined as RBP or retinol concentrations <0.7 and <1.05 μmol/L (32), respectively. Folate deficiency was defined as a plasma or serum folate concentration <10 nmol/L, and vitamin B-12 deficiency was defined as a serum cobalamin concentration <150 pmol/L (33). Folate and vitamin B-12 were measured in the Côte d’Ivoire, Mexico 2012, and US surveys, folate alone was in measured in Georgia, and vitamin B-12 alone was measured in Colombia. Folate and vitamin B-12 concentrations were not corrected for inflammation.

CRP was measured in all surveys, but AGP was only measured in the Cameroon, Côte d’Ivoire, Laos, Liberia, and Papua New Guinea surveys. Because not all surveys measured both CRP and AGP, we classified inflammation status into 2 categories as follows: no inflammation and any inflammation. No inflammation was defined as having both a normal CRP concentration (≤5 mg/L) and a normal AGP concentration (≤1.0 g/L) or, in the absence of an AGP value, having a normal CRP concentration. Survey subjects with no biochemical measure of inflammation were recoded as missing values. Accordingly, ferritin values were also recoded as missing if no data on inflammation was available. BMI (in kg/m^2^) was classified as follows: <18.5 (underweight), 18.5–24.9 (normal), 25.0–29.9 (overweight), and ≥30.0 (obese) (34).

Three household-level factors that were related to water, sanitation, hygiene, and socioeconomic status (SES) were examined as potential covariates of women’s anemia status. Water, sanitation, and hygiene indicators were defined according to UNICEF-WHO guidelines (35) by classifying the household drinking water source as being safe or unsafe and the household sanitation facility as being improved or unimproved. Open defecation was used as a third category of household sanitation and was comprised of households with no sanitation facility or those reporting that defecation is usually conducted in the bush or river. Household SES was defined within each survey with the use of appropriate available data. SES was based on household income in Papua New Guinea, the poverty-index ratio in the United States (36), and an asset score in all other surveys (37). Because of a lack of data on household assets or income, SES could not be calculated for Georgian women. For bivariate analyses, the lowest SES group, such as the lowest 2 quintiles of asset scores, was compared with a reference group of all households with higher SES.

### Statistical analysis

Analyses were conducted with SPSS version 23 software (IBM Corp.) with the complex survey module unless otherwise noted. Stratified sampling was done in all surveys from which data were included in the combined BRINDA data set. As a result, each individual survey data set included standardized sampling weights to correct for an unequal probability of selection within that survey. For pooled analyses, population-based weights were rescaled so that the sum of the weights within each study was proportional to the size of the population represented by the data set (i.e., number of WRA).

Univariate statistics were calculated for each country and infection group to describe the surveyed women’s mean age, mean hemoglobin concentration, and proportions of anemia, micronutrient deficiencies, inflammation, and malaria. Bivariate analyses of the associations between anemia prevalence and demographic and nutrition factors was done for each survey data set and, after categorizing the data sets by infection burden, for each infection group. For each risk factor and respective subgroup, the prevalence of anemia was calculated, and the significance of the difference between anemia prevalence for each factor’s subgroups was judged with the use of Pearson’s chi-square test. *P* < 0.05 was used to determine significance in the bivariate analyses.

Factors with significant associations with anemia in the bivariate analyses for each infection-country grouping were included into logistic regression models for each infection-burden group. Within each geographic grouping, we limited the list of covariates to those that were available for all countries in that group with the exception of Papua New Guinea, which was excluded from the multipredictor models because ferritin was not measured as part of this survey. Each factor was included in the regression models regardless of the factor’s position in hypothesized causal chains; i.e., even if 2 factors lay on the same causal chain (e.g., helminth infection and iron deficiency), both were included in the regression models. Models were constructed with the use of block stepwise regression whereby variables were entered into the model in blocks in order of anticipated importance. The blocks and variables in each block were as follows: *1*) main factors (i.e., iron deficiency and inflammation); *2*) nutrition factors (i.e., BMI and vitamin A, folate, and B-12 deficiencies); *3*) health factors (ever given birth, currently breastfeeding, and number of times pregnant); *4*) environmental factors (i.e., household sanitation and water facilities); and *5*) demographic factors (age group, marital status, SES, rural or urban residence, woman’s educational level, and educational level of household head).

The anticipated importance of risk factors was determined according to the BRINDA anemia framework (11) and the authors’ judgment. Each block of variables was separately added to its respective infection-group model in the order of importance, and variables without marginal significance (*P* > 0.1) were progressively removed from each block. In addition, a categorical variable for each survey in the infection group was included in each model to represent the effects of factors that were not captured by the other explanatory variables. This survey variable was retained in each model irrespective of the significance of the association with anemia. No interaction terms were investigated during the model-building process, and no variables were forced into the model. We examined the association between anemia and iron deficiency with the use of 3 separate calculations as follows: *1*) the prevalence of iron-deficiency anemia or the proportion of all women who had concurrent iron deficiency and anemia; *2*) the proportion of iron-deficient and iron-sufficient women who had anemia; and *3*) the proportion of anemic women who were iron deficient.

## RESULTS

### Population characteristics

Our study sample was restricted to participants with hemoglobin values, which excluded 1.9% of WRA (*n* = 521) from the analysis. WRA who were excluded because of missing hemoglobin values had a younger mean age (27.2 y) than that of women who were included in the analysis (29.9 y) but did not have substantially different SES (data not shown). As shown in **Table 1**, 27,018 WRA had valid age and hemoglobin measurements. At the country level, the mean age of the WRA ranged from 27 to 34 y, and WRA in high-infection and moderate-infection countries were slightly younger than were WRA in low-infection countries. The minimum and maximum ages in all surveys were 15 and 49 y except for in the Cameroon and Côte d’Ivoire surveys in which the maximum age was 48 y.

**TABLE 1:** Age, hemoglobin concentrations, and anemia prevalence in nonpregnant women of reproductive age by country and category of infection burden: the BRINDA project^1^

	*n*	Age,^2^ y	Hemoglobin,^2^ g/L	Severe anemia, %	Moderate anemia, %	Mild anemia, %	Any anemia, %
Survey							
Cameroon	775	27.2 (26.5, 27.8)	122.6 (120.8, 124.3)	1.3 (0.6, 2.5)	15.7 (12.6, 19.4)	19.3 (16.6, 22.4)	36.3 (31.9, 41.0)
Colombia	9678	29.1 (28.8, 29.3)	141.6 (141.0, 142.1)	0.1 (0.1, 0.3)	4.8 (4.3, 5.3)	3.0 (2.6, 3.6)	8.0 (7.3, 8.7)
Côte d’Ivoire	850	27.6 (26.9, 28.3)	118.6 (117.4, 119.9)	2.0 (1.3, 3.1)	23.7 (20.7, 26.9)	24.2 (21.5, 27.1)	49.9 (46.4, 53.3)
Georgia	1711	32.6 (32.0, 33.1)	129.7 (128.0, 131.3)	1.1 (0.6, 1.8)	8.2 (6.7, 10.1)	14.1 (11.9, 16.5)	23.3 (20.4, 26.6)
Laos	823	29.3 (28.6, 30.0)	122.6 (120.3, 124.9)	2.4 (1.5, 3.9)	16.0 (11.8, 21.3)	17.6 (14.5, 21.2)	36.0 (30.6, 41.9)
Liberia	1971	28.6 (28.1, 29.1)	123.7 (122.6, 124.7)	0.2 (0.1, 0.6)	11.3 (9.4, 13.5)	21.7 (19.4, 24.2)	33.2 (29.7, 37.0)
Mexico 2006	3050	31.3 (30.7, 31.9)	136.4 (135.2, 137.5)	0.1 (<0.1, 0.2)	5.2 (4.0, 6.7)	8.6 (6.9, 10.8)	13.9 (11.8, 16.3)
Mexico 2012	4174	32.1 (31.7, 32.5)	135.2 (134.6, 135.9)	0.6 (0.3, 1.1)	4.8 (4.1, 5.7)	7.5 (6.4, 8.7)	12.9 (11.6, 14.3)
Papua New Guinea	760	29.2 (28.5, 29.9)	125.7 (123.1, 128.3)	2.3 (1.5, 3.6)	15.7 (12.0, 20.3)	17.0 (13.6, 21.0)	35.1 (29.0, 41.7)
United States	3226	33.5 (32.9, 34.0)	135.5 (134.5, 136.4)	0.1 (<0.1, 0.2)	2.6 (2.0, 3.4)	4.0 (3.2, 5.0)	6.7 (5.5, 8.0)
Infection burden							
Low	4937	33.5 (33.0, 33.9)	135.4 (134.4, 136.3)	0.1 (<0.1, 0.2)	2.7 (2.1, 3.3)	4.1 (3.3, 5.1)	6.9 (5.9, 8.1)
Moderate	16,902	31.2 (31.0, 31.5)	136.8 (136.3, 137.3)	0.3 (0.2, 0.5)	5.0 (4.4, 5.6)	7.1 (6.3, 8.1)	12.4 (11.4, 13.5)
High	5179	27.9 (27.6, 28.2)	121.7 (120.9, 122.5)	1.7 (1.3, 2.2)	18.1 (16.4, 19.9)	20.7 (19.2, 22.2)	40.5 (38.3, 42.6)

1 Severe anemia was defined as a hemoglobin concentration <80 g/L; moderate anemia was defined as a hemoglobin concentration ≥80 and <110 g/L; mild anemia was defined as a hemoglobin concentration ≥110 and <120 g/L; and any anemia was defined as a hemoglobin concentration <120 g/L. Hemoglobin concentrations were adjusted for altitude and the intensity of cigarette smoking in the Colombia, Georgia, Papua New Guinea, Mexico 2006, and Mexico 2012 surveys. In Laos, hemoglobin was only adjusted for altitude, and in the United States, hemoglobin was only adjusted for smoking. No adjustments to hemoglobin were made in the Côte d’Ivoire, Cameroon, and Liberia surveys. Countries were categorized by infection burden as follows—low: Georgia and the United States; moderate: Colombia and Mexico (2006 and 2012); high: Cameroon, Côte d’Ivoire, Liberia, Laos, and Papua New Guinea. BRINDA, Biomarkers Reflecting Inflammation and Nutritional Determinants of Anemia.

2 Values are means (95% CIs).

Mean hemoglobin concentrations ranged from 119 g/L in Côte d’Ivoire to 142 g/L in Colombia and were noticeably lower in high-infection countries than in moderate- and low-infection countries. Severe anemia was relatively rare with a prevalence of <2.5% in all countries. The prevalence of moderate anemia ranged from ∼3% in the United States to nearly 24% in Cote d’Ivoire and was >3 times higher in high-infection countries than in moderate- and low-infection countries. Similarly, the range in the prevalence of mild anemia was large with a prevalence that was 3–5 times higher in high-infection countries than in moderate- and low-infection countries. Mild anemia accounted for >50% of all anemia shown in all infection groups. Anemia was a severe public health problem in Côte d’Ivoire, a moderate public health problem in Cameroon, Georgia, Laos, Liberia, and Papua New Guinea, and a mild public health problem in Colombia, Mexico, and the United States. The prevalence of any anemia in high-infection countries was 28 and 34 percentage points higher than in moderate- and low-infection countries, respectively.

**FIGURE 1:**
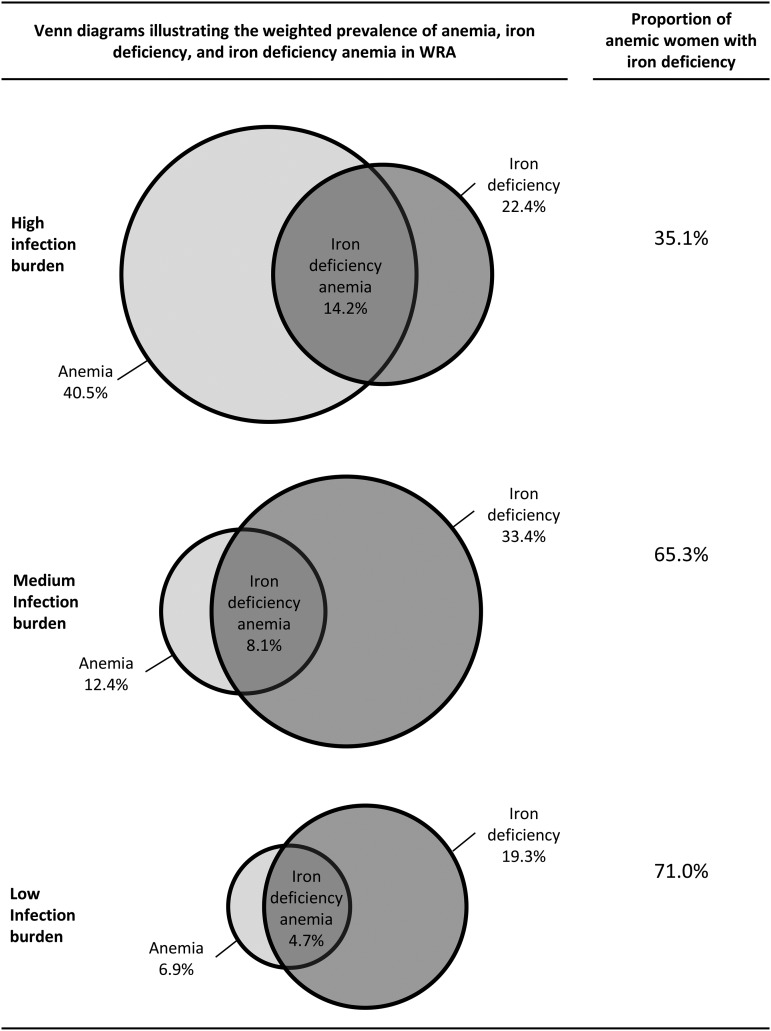
Venn diagrams illustrating the weighted prevalences of iron deficiency, anemia, and iron-deficiency anemia and proportions of anemic women with iron deficiency in nonpregnant WRA by category of infection burden: Biomarkers Reflecting Inflammation and Nutritional Determinants of Anemia (BRINDA) project. Iron deficiency was defined as an inflammation-adjusted ferritin concentration <15 μg/L. Anemia was defined as a hemoglobin concentration <120 g/L. Iron-deficiency anemia was defined as a hemoglobin concentration <120 g/L and inflammation-adjusted ferritin concentration <15 μg/L. Countries were categorized by infection burden as follows—low: Georgia and the United States; moderate: Colombia and Mexico (2006 and 2012); and high: Cameroon, Côte d’Ivoire, Liberia, and Laos. WRA, women of reproductive age.

The estimated prevalence of iron deficiency on the basis of inflammation-adjusted ferritin values ranged from 19% to 38% in all countries except in Georgia where the prevalence was <2% (**Table 2**). The prevalence of iron-deficiency anemia was >10% in 4 countries (Cameroon, Côte d’Ivoire, Laos, and Liberia) in the high-infection group. The adjustment of ferritin for inflammation increased the proportion of women with iron deficiency and iron-deficiency anemia in all countries and infection groups. The largest change in iron deficiency was observed in the moderate-infection group where the prevalence increased by 8.4 percentage points. Despite this large increase in the prevalence of iron deficiency, the largest change in iron-deficiency anemia was observed in the high-infection group where the prevalence increased by 3.5 percentage points (**Figure 1**, **Supplemental Figure 2**).

**TABLE 2:** Prevalence of micronutrient deficiencies, inflammation, and malaria in nonpregnant women of reproductive age by country and category of infection burden: the BRINDA project^1^

	*n*	Iron deficiency, %	Iron-deficiency anemia, %	Vitamin A insufficiency, %	Folate deficiency, %	Vitamin B-12 deficiency, %	Inflammation, %	Malaria, %
Survey								
Cameroon	775	19.1 (16.0, 22.8)	13.0 (10.3, 16.3)	16.4 (13.7, 19.4)	—	—	20.3 (17.2, 23.7)	14.8 (11.5, 18.9)
Colombia	9678	25.0 (23.8, 26.2)	4.5 (4.0, 5.1)	—	—	15.2 (3.2, 49.7)	21.8 (20.6, 23.0)	—
Côte d’Ivoire	850	21.0 (18.0, 24.3)	14.4 (12.3, 16.7)	13.2 (10.6, 16.3)	86.4 (83.0, 89.1)	18.0 (12.3, 25.7)	33.5 (29.8, 37.4)	4.9 (3.5, 6.7)
Georgia	1711	1.4 (0.9, 2.3)	0.7 (0.4, 1.4)	—	80.5 (74.8, 85.1)	—	29.3 (26.5, 32.4)	—
Laos	823	34.4 (28.3, 41.2)	17.4 (13.3, 22.3)	—	—	—	13.9 (11.3, 17.1)	—
Liberia	1971	27.1 (24.2, 30.1)	14.3 (12.3, 16.5)	20.4 (18.0, 23.1)	—	—	18.4 (16.2, 20.7)	17.7 (15.3, 20.5)
Mexico 2006	3050	32.5 (29.6, 35.5)	8.3 (6.8, 10.1)	—	—	—	24.2 (21.8, 26.8)	—
Mexico 2012	4174	37.7 (34.6, 41.0)	9.4 (7.5, 11.9)	—	2.5 (1.8, 3.3)	1.7 (1.2, 2.3)	20.7 (18.3, 23.3)	—
Papua New Guinea	749	—	—	7.7 (5.7, 10.3)	—	—	24.8 (21.3, 28.8)	—
United States	3226	19.5 (17.7, 21.6)	5.0 (4.2, 5.9)	3.0 (2.3, 3.7)	2.8 (2.0, 3.8)	2.8 (2.0, 3.9)	25.6 (23.6, 27.7)	—
Infection burden								
Low	4937	19.3 (17.4, 21.3)	4.9 (4.2, 5.8)	3.0 (2.3, 3.7)	3.1 (2.3, 4.1)	2.8 (2.0, 3.9)	25.7 (23.7, 27.7)	—
Moderate	16902	33.4 (31.6, 35.2)	8.1 (7.1, 9.3)	—	2.5 (1.8, 3.3)	5.8 (1.8, 17.2)	22.3 (20.9, 23.8)	—
High	5168	22.4 (20.4, 24.5)	14.2 (12.6, 15.9)	14.4 (12.8, 16.1)	86.4 (83.0, 89.1)	18.0 (12.3, 25.7)	24.4 (22.5, 26.3)	10.8 (9.1, 12.8)

1 Values in parentheses are 95% CIs. Iron deficiency was defined as an inflammation-adjusted ferritin concentration <15 μg/L. Iron-deficiency anemia was defined as a hemoglobin concentration <120 g/L and an inflammation-adjusted ferritin concentration <15 μg/L. Vitamin A insufficiency was defined as a retinol-binding protein or retinol concentration <1.05 μmol/L. Folate deficiency was defined as a folate concentration <10 nmol/L. Vitamin B-12 deficiency was defined as a vitamin B-12 concentration <150 pmol/L. Inflammation was defined as a CRP concentration >5 mg/L or AGP concentration >1 g/L (only CRP data were available for Georgia, Mexico, and the United States). Sample sizes varied slightly by biomarker per country and a few notable differences were as follows—folate in Georgia: *n* = 401; vitamin B-12 in Côte d’Ivoire: *n* = 398; and vitamin B-12 in Colombia: *n* = 637. Countries were categorized by infection burden as follows—low: Georgia and the United States; moderate: Colombia and Mexico (2006 and 2012); high: Cameroon, Côte d’Ivoire, Liberia, Laos, and Papua New Guinea. AGP, α-1-acid glycoprotein; BRINDA, Biomarkers Reflecting Inflammation and Nutritional Determinants of Anemia; CRP, C-reactive protein.

In the 5 countries where RBP or retinol was measured in WRA, the prevalence of vitamin A deficiency was ≤2% (data not shown). The prevalence of vitamin A insufficiency ranged from 20% in Liberia to 3% in the United States and was ∼14% in high-infection countries with data (Table 2). The prevalence of any inflammation ranged from 14% in Laos to 34% in Côte d’Ivoire and did not substantially differ in the infection groups. Cameroon and Liberia had a similar prevalence of malaria, but a markedly lower prevalence was shown in Côte d’Ivoire where only current malaria was assessed with the use of microscopy. Serum or plasma folate was only measured in a few countries. The prevalence of folate deficiency was >80% in both Côte d’Ivoire and Georgia but <3% in Mexico and the United States. Similar to folate deficiency, the prevalence of vitamin B-12 deficiency was very low (<3%) in both Mexico and the United States but was much higher (∼15%) in Côte d’Ivoire and Colombia.

### Bivariate associations between anemia and iron and vitamin A deficiencies

Only in the high-infection group was the anemia prevalence greater than the iron-deficiency prevalence. In the groups with moderate and low infection burdens, the prevalence of iron deficiency was nearly 3 times greater than the respective group's anemia prevalence. Despite the relatively higher prevalence of iron deficiency in the high-infection group, the contribution of iron deficiency to anemia, as measured via the proportion of anemic women with concurrent iron deficiency, increased with a declining infection burden from 35.1% in the high-infection group to 65.3% in the moderate-infection group, to 71.0% in the-low infection group (Figure 1). When calculated with the use of unadjusted ferritin concentrations, the proportion of anemic women with iron deficiency was markedly lower as 26.4%, 48.8%, and 46.4% in the groups with low, moderate, and high infection burdens, respectively (Supplemental Figure 2).

Anemia was most consistently associated with iron deficiency and vitamin A insufficiency (**Table 3**). The prevalence of anemia was higher in iron-deficient women in all countries and all infection groups. For the high-, moderate-, and low-infection groups, the anemia prevalence of iron-deficient women was 1.8, 3.7, and 10.7 times higher, respectively, than that of their iron-replete counterparts (**Supplemental Table 2**).

**TABLE 3:** Prevalence of anemia by micronutrient deficiencies, inflammation, and health conditions in nonpregnant women of reproductive age by country and by infection burden: the BRINDA project^1^

	Iron		Vitamin A		Folate		Vitamin B-12		Inflammation		BMI, kg/m^2^				Malaria	
Survey	Deficient	Sufficient	Insufficient	Sufficient	Deficient	Sufficient	Deficient	Sufficient	Yes	No	<18.5	18.5–24.9	25.0–29.9	≥30	Yes	No
High infection burden, %	63.3	34.8***	56.5	38.5***	50.8	47.2	55.9	47.8	52.9	36.5***	49.2	43.7	32.8	28.0***	46.6	41.4
Cameroon	68.6	28.1***	52.7	32.6***	NA	NA	NA	NA	51.3	32.0***	46.6	41.1	25.2	24.4***	45.5	34.7*
Côte d’Ivoire	68.5	45.5***	63.6	48.3*	50.8	47.2	55.9	47.8	58.4	46.3**	55.5	50.7	49.8	35.0	57.5	49.5
Laos	50.4	28.4***	NA	NA	NA	NA	NA	NA	42.2	34.9	39.7	36.8	28.1	27.6	NA	NA
Liberia	52.7	26.2***	47.0	29.9***	NA	NA	NA	NA	46.9	30.3***	NA	NA	NA	NA	37.2	32.6
Papua New Guinea	NA	NA	63.7	33.5***	NA	NA	NA	NA	44.6	32.9*	56	38.1	20.3	23.8***	NA	NA
Moderate infection burden, %	24.4	6.7***	NA	NA	19.0	12.8	3.6	11.1	14	12.1	9.9	11.8	13.0	12.6	NA	NA
Colombia	18.1	4.2***	NA	NA	NA	NA	1.7	6.1	8.5	7.5	9.2	7.4	8.0	8.8	NA	NA
Mexico 2006	25.8	8.4***	NA	NA	NA	NA	NA	NA	14.8	13.9	12.5	13.6	14.9	12.5	NA	NA
Mexico 2012	24.6	6.6***	NA	NA	19.0	12.8	11.3	13.0	15.7	12.4	8.1	12.8	13.0	13.3	NA	NA
Low infection burden, %	25.6	2.4***	25.2	6.2***	10.8	6.6	7.3	6.7	8.8	6.2*	3.6	5.1	7.3	9.2**	NA	NA
Georgia	51.2	23.2**	NA	NA	23.5	19.1	NA	NA	27.8	21.8*	31.3	23.7	21.0	23.0	NA	NA
United States	25.5	2.0***	25.2	6.2***	9.6	6.6	7.3	6.7	8.5	6.0**	3.0	4.7	7.0	9.1**	NA	NA

1 All values are means. Iron deficiency was defined as an inflammation-adjusted ferritin concentration <15 μg/L. Vitamin A insufficiency was defined as an unadjusted retinol-binding protein or retinol concentration <1.05 μmol/L. Folate deficiency was defined as a serum or plasma folate concentration <10 nmol/L. Vitamin B-12 deficiency was defined as a serum or plasma vitamin B-12 concentration <150 pmol/L. Any inflammation was defined as a C-reactive protein concentration >5 mg/L or α-1-acid glycoprotein concentration >1 g/L. *^,^**^,^***Pearson’s chi-square test: *P < 0.05, **P < 0.01, ***P < 0.001. BRINDA, Biomarkers Reflecting Inflammation and Nutritional Determinants of Anemia; NA, not available

The anemia prevalence in WRA with vitamin A insufficiency was higher in Côte d’Ivoire, with a greater significance (*P* < 0.001) in Cameroon, Liberia, Papua New Guinea, and the United States. Noteworthy associations (*P* < 0.05) between anemia and vitamin A deficiency were shown in 3 of 5 countries (i.e., Liberia, Papua New Guinea, and the United States) and in the groups with high and low infection burdens (data not shown).

### Bivariate associations between anemia and folate and vitamin B-12 deficiencies, inflammation, and BMI

The prevalence of anemia did not significantly differ by folate or vitamin B-12 status. Notably, small sample sizes for folate status in Georgia (*n* = 401) and for vitamin B-12 in Côte d’Ivoire (*n* = 398) and Colombia (*n* = 637) may have affected the sensitivity of the analyses in those countries. Mixed results were observed for any inflammation; the prevalence of anemia in WRA with inflammation was significantly higher in all countries except Colombia, Laos, and Mexico. Significant differences in anemia prevalence by inflammation status were shown in the groups with high and low infection burdens but not in the groups with a moderate infection burden.

Mixed associations between anemia and BMI were also observed with significant differences in high– and low–infection-burden groups but not in the moderate–infection-burden group (Table 3). Notably, high- and low-infection groups had opposite trends in the prevalence of anemia and BMI whereby, in high–infection-burden group, anemia prevalence decreased with increasing BMI, whereas anemia prevalence and BMI increased in tandem in the low-infection group. Although the prevalence of anemia was consistently higher in WRA with malaria, these differences were NS in 2 of 3 countries or in the pooled analyses.

### Bivariate associations between anemia and demographic factors

The demographic factors that were explored varied by survey, but SES showed a consistent and mostly significant association with anemia prevalence (**Table 4**). For all countries except Cameroon, Liberia, and Papua New Guinea, SES was significantly inversely associated with anemia, and significant differences were observed in all pooled infection groups. Moreover, a clear trend in anemia was observed with prevalence consistently decreasing from the low- to high-socioeconomic groups. WRA in different age groups showed significant differences in the prevalence of anemia in some countries and infection groups; however, there was only a clear trend in the Colombia, Liberia, and Mexico (2012) surveys and moderate–infection-burden group. In the Colombia and Mexico 2012 surveys and the moderate–infection-burden group, the prevalence of anemia increased with age, whereas in Liberia, the prevalence of anemia decreased with age.

**TABLE 4:** Prevalence of anemia by sociodemographic factors in nonpregnant women of reproductive age by country and by infection burden: the BRINDA project^1^

	Age, y				Residence		Socioeconomic status			Sanitation facilities			Water source		Educational attainment			
	15–20	21–30	31–40	41–49	Rural	Urban	Low	Medium	High	None	Unimproved	Improved	Unimproved	Improved	None	Primary	Secondary	University or trade
Survey, %																		
Cameroon	34.8	37.5	34.9	33.3	36.1	36.4	40.2	33.1	34.7	61.4	38.2	34.8	40.7	35.0	44.6	34.3	31.8	41.6
Colombia	59.5	47.3	50.1	48.5	55.2	45.4**	56.3	48.3	41.2**	52.9	53.1	48.1	58.9	48.3	52.4	47.5	51.1	36.2
Côte d’Ivoire	6.2	6.4	9.5	10.7***	9.1	7.6	9.6	7.3	6.2***	6.4	11.2	8.7	7.3	8.8	6.9	8.6	9.1	9.1
Georgia	26.6	18.8	26.5	23.7	24.5	22.2	NA	NA	NA	NA	NA	NA	NA	NA	29.6	25.9	23.1	23.5
Laos	40.9	35.2	35.9	32.4	40.0	27.7*	47.2	31.1	26.7***	46.5	27.7	26.4***	36.5	35.7	45.1	37.6	28.4	NCs*
Liberia	40.2	33.2	31.7	26.6*	33.1	33.6	30.7	35.3	33.0	33.7	31.6	35.0	22.8	35.2***	29.0	35.6	38.8	28.3**
Mexico 2006	9.8	9.6	14.4	16.0**	12.2	13.2	14.6	13.1	9.5*	NA	NA	NA	NA	NA	NA	NA	NA	NA
Mexico 2012	12.3	14.6	13.1	15.5	17.0	12.7	15.5	14.3	7.6*	17.1	13.3	13.9	NA	NA	NA	NA	NA	NA
Papua New Guinea	34.2	34.5	36.0	35.7	38.7	21.5**	41.8	32.0	29.2	NA	NA	NA	NA	NA	35.9	35.9	34.5	NCs
United States	6.6	4.5	5.7	9.0**	NA	NA	10.0	6.6	4.1***	NA	NA	NA	NA	NA	NCs	NCs	7.0	6.1
Infection burden, %																		
Low	6.8	4.8	6.0	9.2**	24.5	22.2	10.0	6.6	4.1***	NA	NA	NA	NA	NA	29.6	25.9	7.2	6.3
Moderate	9.7	11.0	13.3	14.9*	13.6	11.9	14.2	12.5	8.4***	14.6	13.3	13.1	7.3	8.8	6.9	8.6	9.1	9.1
High	44.6	40.1	40.0	37.8	42.4	38.4	45.8	38.4	34.8***	49.3	38.9	39.3**	41.9	41.1	46.5	38.4	35.4	36.8***

1 All values are means. Household sanitation and drinking water source were defined according to the WHO/UNICEF Joint Monitoring Program for water supply and sanitation. Sanitation: none = open defecation. Countries were categorized by infection burden as follows—low: Georgia and the United States; moderate: Colombia and Mexico (2006 and 2012); high: Cameroon, Côte d’Ivoire, Liberia, Laos, and Papua New Guinea. *^,^**^,^***Pearson’s chi-square *P* values indicate that the proportion in at least one subgroup is significantly different from the values in the other subgroups: *P < 0.05, **P < 0.01, ***P < 0.001. BRINDA, Biomarkers Reflecting Inflammation and Nutritional Determinants of Anemia; NA, not available; NC, no case in the subgroup.

In Cote d’Ivoire, Laos, and Papua New Guinea, rural residents had a higher prevalence of anemia than did urban residents. Significant associations between household sanitation and anemia were observed in Laos only with the anemia prevalence in WRA residing in households practicing open defection being notably higher than in households with the use of improved or unimproved latrines. This strong country-level association was likely responsible for the significant difference that was observed in the high-infection group. Significant associations between the quality of the household water source and the prevalence of anemia were observed in Liberia only where the prevalence of anemia was lower in WRA who were residing in households that consumed water from unimproved sources. The water source was not significantly associated with anemia in the pooled analyses. Only in Laos was there a significant association and a clear trend between education status and the prevalence of anemia. Education status and anemia were associated with significance in the high-infection group, and the anemia prevalence in WRA with no formal education was ∼10 percentage points higher than in WRA in other education subgroups.

### Multivariable models of associations with anemia

Results from the pooled multivariable analyses for each infection group are presented in **Table 5**. In the high-infection group, independent predictors of anemia included iron deficiency, inflammation, and vitamin A insufficiency. All other potential predictors were removed from the model because they were NS. The odds of anemia in iron-deficient women was 3.7 times higher than that of iron-replete women. In addition, women with inflammation (elevated CRP or AGP) had 1.9 times the odds of being anemic than that of women with no inflammation. Similarly, the odds of anemia in vitamin A–insufficient women was 1.7 times higher than in vitamin A–replete women. These results were all highly significant (*P* < 0.001).

**TABLE 5:** Logistic regression models of factors associated with anemia in nonpregnant women of reproductive age by category of infection burden: the BRINDA project^1^

	Low infection burden		Moderate infection burden		High infection burden	
Variable	OR (95% CI)	*P*	OR (95% CI)	*P*	OR (95% CI)	*P*
Iron deficiency	16.8 (11.5, 24.6)	<0.001	4.3 (3.4, 5.4)	<0.001	3.7 (2.9, 4.8)	<0.001
Inflammation	1.5 (1.01, 2.1)	0.046	—		1.9 (1.5, 2.4)	<0.001
Vitamin A insufficiency	—		—		1.7 (1.2, 2.3)	<0.001
Age group, y						
15–19	Reference	<0.01	Reference	0.015	—	
20–29	0.78 (0.45, 1.3)		0.97 (0.6, 1.6)		—	
30–39	1.1 (0.68, 1.8)		1.3 (0.89, 1.8)		—	
40–49	1.7 (1.05, 2.6)		1.5 (1.1, 2.2)		—	
Socioeconomic status						
Low	—		1.8 (1.4, 2.4)	<0.001	—	
Moderate	—		1.6 (1.2, 2.2)		—	
High	—		Reference		—	

1 Anemia status (yes or no) was used as the outcome variable in all models. Countries were categorized by infection burden as follows—low: Georgia and the United States; moderate: Colombia and Mexico (2006 and 2012); high: Cameroon, Côte d’Ivoire, Liberia, and Laos. As the Papua New Guinea survey did not measure ferritin, it was not included in the high infection burden model. Iron deficiency was defined as an inflammation-adjusted ferritin concentration <15 mg/L. Inflammation was defined as a C-reactive protein concentration >5 mg/L or α-1-acid glycoprotein concentration >1 g/L. Vitamin A insufficiency was defined as an unadjusted retinol-binding protein or retinol concentration <1.05 μmol/L. CIs were adjusted for survey weights that were proportional to the size of the target population in each country. Em dashes denote variables that were not included in the final regression models because of a lack of significance. Other variables (i.e., BMI, folate and vitamin B-12 deficiencies, ever given birth, currently breastfeeding, number of times pregnant, household sanitation, household water facilities, marital status, rural or urban residence, woman’s education level, and education level of the household head) were removed from the various models because of a lack of significance. BRINDA, Biomarkers Reflecting Inflammation and Nutritional Determinants of Anemia.

In moderate-infection countries, iron deficiency, age, and SES were significant predictors of anemia. Other predictors that were NS were removed from the model. Iron-deficient women had 4.3 times the odds of being anemic than that of iron-replete women (*P* < 0.001). Compared with the reference group (i.e., high SES), the odds of anemia were significantly higher in the low-socioeconomic group (OR 1.8; 95% CI: 1.4, 2.4) and moderate-socioeconomic group (OR 1.6; 95% CI: 1.2, 2.2) (*P* < 0.001). Regarding age, the odds of being anemic were 50% higher in women aged 40–49 y than in women aged 15–19 y.

In the low–infection-burden model, iron deficiency, inflammation, and age were significant predictors of anemia status. Other factors were removed from the model because they were NS. Iron-deficient women had nearly 17 times the odds (95% CI: 11.5, 24.6) of being anemic than did their iron-replete counterparts (*P* < 0.001). The odds of anemia in women with inflammation were 50% greater (OR 1.5, 95% CI: 1.006. 2.1) than in women without inflammation (*P* < 0.05). As regards age, the odds of anemia in women aged 20–29 and 30–39 y were similar to those of the reference population (i.e., women aged 15–19 y). Only women aged 40–49 y had significantly higher odds of anemia than those in the reference group.

## DISCUSSION

Our results on factors associated with anemia were based on the analysis of individual-level biomarker data and, as such, differ from other recently-published multicountry analyses by Kassebaum et al. (13) and Petry et al. (25). Kassebaum et al. (13) estimated the contribution of numerous risk factors to anemia by estimating the “hemoglobin shift” or impact of a risk factor on hemoglobin concentrations; systematic reviews and meta-analyses were used to estimate the hemoglobin shift for each risk factor. The authors showed that iron deficiency was the single greatest contributor to anemia globally but that other factors (e.g., malaria and helminths) also contributed substantially to the anemia burden in some regions. Although the authors used a rigorous approach, their model could not take into account the severity of iron deficiency in each country. Contrarily, the analysis by Petry et al. (25) focused solely on the contribution of iron deficiency to anemia. By conducting a meta-analyses of 23 national-level data sets containing results of anemia and iron deficiency, they showed that, in nonpregnant WRA, 37% of anemia was attributable to iron deficiency, and the proportion of anemia that was attributable to iron deficiency was lowest (∼18%) in women in countries where anemia was a severe public health problem. Similar to the findings by Kassebaum et al. (13), there was considerable variability in the proportion of anemia that was attributable to iron deficiency by region.

Our findings support those of Kassebaum et al. (13) and Petry et al. (25) and confirm that the assumption that 50% of anemia is attributable to iron deficiency cannot be consistently applied to WRA. Furthermore, our analysis suggests that this approximation may overestimate the proportion of anemia that is attributable to iron deficiency in countries with a high infection burden and underestimate the same in countries with a lower infection burden. In addition, the overall prevalence of anemia and iron deficiency and their ratio to each other varied dramatically by infection group, thereby strongly suggesting that the anemia and iron-deficiency burden is not uniform and varies by setting.

Individual studies in other countries with high infection burdens have shown similar findings. In Sierra Leone (38), 45% of nonpregnant WRA were anemic, but only 8% and 6% of them had iron deficiency and iron-deficiency anemia, respectively. Despite the small prevalence of iron deficiency in Sierra Leonean women, iron deficiency was significantly associated with anemia in multivariate models but only contributed to 11% of anemia. In the Democratic Republic of the Congo, Harvey-Leeson et al. (19) showed a moderate prevalence of anemia in the South Kivu (17%) and Kongo Central (32%) provinces but only 5% of iron deficiency and <1% of iron-deficiency anemia in both provinces. In rural Bangladesh, Merrill et al. (39) observed a high prevalence of anemia (57%) and complete absence (0%) of iron deficiency and speculated that anemia was most attributable to increased parity, and high body iron stores were due to high levels of iron in the groundwater.

An understanding of the proportion of anemia that is attributable to iron deficiency is particularly relevant to the design and implementation of public health programs such as the fortification of staple foods with iron and the promotion and distribution of iron supplements. The fortification of cereal grains with iron is currently mandatory in 85 countries (40) with wheat, maize, and rice being the most widely fortified food grains. Staple condiments, such as soy sauce and bouillon cubes (41), are also increasingly fortified with iron, and home-fortification products that contain iron are also designed specifically for pregnant women (42). Accompanying this suite of fortified foods are prophylactic iron supplements, which the WHO recently recommended for “menstruating adult women and adolescent girls” in settings where anemia is >40% (43).

Fortification and supplementation interventions often have the explicit aim to reduce anemia, but data on the etiology of anemia are lacking for many countries. Moreover, the use of anemia prevalence as a criterion for implementing iron interventions may not be the most effective approach particularly where the infection burden is high. To illustrate, a recent systematic review of flour-fortification–effectiveness studies showed that flour-fortification programs (all containing iron fortificants) more consistently reduced iron deficiency than anemia (44). In contrast, a systematic review of daily iron supplementation showed consistent associations with improved hemoglobin concentrations and reductions in anemia (45). Both reviews noted that there are only limited data available on the effectiveness of iron interventions and suggested that, particularly for fortification programs, programs may be more effective at reducing iron deficiency than anemia.

In our analysis, vitamin A status was only available for 5 countries, 4 of which (Cameroon, Côte d'Ivoire, Liberia, and Papua New Guinea) were included in the high-infection group. Thus, there were insufficient data to measure this association in the moderate- and low-infection groups. Nonetheless, for the high-infection group, our findings suggest that vitamin A insufficiency independently contributes to anemia in women. Radhika et al. (46) showed similar results in pregnant women from India with a significant association between vitamin A deficiency (serum retinol concentration <1.05 μmol/L) and anemia.

Inflammation, as defined by elevated CRP or AGP, was repeatedly associated with anemia in our analysis. The inflammatory response can induce anemia via the downregulation of iron absorption and erythropoiesis (47); independent associations between inflammation and low hemoglobin concentrations have been reported in Denmark (48), Sierra Leone (38), and the United States (49). Despite our separation of countries into groups on the basis of infection burden, we showed a similar inflammation prevalence (22–26%) in the infection groups. Inflammation has multiple causes, and significant associations between inflammation and anemia in both the high- and low-infection groups may have reflected inflammation stemming from different causes. As part of this supplement, Merrill et al. (50) showed that a positive malaria status was significantly associated with both acute inflammation (i.e., elevated CRP) and chronic inflammation (i.e., elevated AGP) in WRA in Cameroon, Côte d'Ivoire, and Liberia. They also showed that the associations between acute inflammation and obesity were largely consistent; the odds of elevated CRP were 3–8 times higher in obese women than in normal-weight women in 7 of 9 countries.

Although we did not find an association between inflammation and anemia in the moderate-infection group, note that this group was comprised of only the Colombia, Mexico 2006, and Mexico 2012 surveys, which did not include AGP as a measure of chronic inflammation. The absence of AGP as an indicator could have resulted in a weaker relation between inflammation and anemia in this group.

A similar pattern of associations was shown between age and anemia in the moderate- and low-infection groups with the prevalence of anemia being highest in women aged 40–49 y. These finding are somewhat different to anemia-age trends that were calculated by Kassebaum et al. (13) who observed a steady decline in anemia prevalence in women after ∼20 y of age. Sekhar et al. (51) showed that the risk factors for iron-deficiency anemia differed in adolescent girls (aged 12–19 y) and adult women (aged 21–49 y) with the use of contraception reducing risk in adolescent girls and the number of live births being associated with increasing risk in older women.

The lack of an association between malaria and anemia in the multivariate model may have been due to the fact that malaria was not assessed in a standardized manner in the 3 West African surveys. In addition, because of the significant association between malaria and inflammation (50), inflammation may have served as a proxy measure of malaria in the 3 surveys from West Africa. We also showed no association between anemia and folate and vitamin B-12 deficiencies, which is a finding that is supported by Metz (52), who conducted a review of studies that examined the hematologic effects of folate and vitamin B-12 deficiencies. Folate and vitamin B-12 were measured in 50% of the surveys, and small sample sizes in a few surveys may have affected the sensitivity of the analysis.

Although the interpretation of ferritin concentrations is complicated by the presence of inflammation, our analysis took advantage of newer methods for mathematically adjusting these indicators for inflammation, namely, the use of a regression approach rather than the application of correction factors on the basis of infection categories as proposed previously by Thurnham et al. (29, 30). The regression approach resulted in a greater reduction in the prevalence of iron deficiency than shown with correction-factor approaches; this difference in iron assessment may explain some inconsistencies with the results of other studies. Note that the regression approach was not used to adjust RBP and retinol because the BRINDA project showed inconsistent associations between inflammation and vitamin A–status markers in women (31). Although the unadjusted RBP and retinol values appeared to not have been affected by inflammation, even a weak or nonlinear association between inflammation and vitamin A status could have affected the associations shown between vitamin A insufficiency and anemia.

Our analysis has some notable limitations. First, data were cross-sectional, which prevented any temporal analysis of causation. Second, the multivariate approach that we used did not take into account the relations between the predictor variables; as such, future analyses should explore statistical methods (e.g., a path analysis) that permit the estimation of the direct and indirect influences of predictors on anemia status. Third, the BRINDA data set used only hemoglobin to measure anemia and did not contain other hematologic measures (e.g., mean corpuscular volume) that can be used to determine microcytic or macrocytic anemia and could have better elucidated the interrelation between the risk factors for anemia (53). Fourth, not all data sets contained measures of AGP, which compromised the measurement of inflammation. Fifth, the BRINDA data set did not include other potential determinants of anemia such as hemoglobinopathies, helminth burden, and HIV status, which may have a role in certain settings (12), and not all data sets contained data on folate and vitamin B-12 deficiencies. Sixth, the BRINDA project only contained a small number of data sets that were subsequently classified into the high-, moderate-, and low-infection groups. The small number of studies in these groups limits the generalizability of our findings, particularly for the pooled analyses of the low-infection group, because the weights that were based on the proportional population size of each country limited the contribution of Georgia because of its relatively small population size. Last, the grouping of countries by infection group may represent other factor differences besides infection, including the SES of the country, dietary patterns, and access to health care.

In conclusion, particularly in countries where the prevalence of anemia is a moderate or severe public health problem, more data elucidating the etiology of anemia are needed. The data that were used in our analysis are relatively rare and greater efforts are needed to collect and compile similar data in other countries that experience high anemia prevalence. A context-specific understanding of the determinants of anemia is needed to design and evaluate national health programs that aim to reduce anemia.
